# Clinical workflow for reirradiation: national consensus recommendations on imaging, treatment **planning**, dose accumulation, and treatment delivery

**DOI:** 10.2340/1651-226X.2025.43567

**Published:** 2025-07-24

**Authors:** Laura P. Kaplan, Rebecca J. Tobin, Ane L. Appelt, Eliana Vasquez Osorio, Isak Wahlstedt, Rasmus L Christiansen, Martin S. Nielsen, Laura A. Rechner, Simon N. Thomsen, Mikkel D. Lund, Kenneth Jensen, Camilla Kronborg, Lone Hoffmann

**Affiliations:** a Department of Oncology and Palliative Units, Zealand University Hospital, Næstved, Denmark; b Department of Oncology, Centre for Cancer and Organ Diseases, Copenhagen University Hospital – Rigshospitalet, Copenhagen, Denmark; c Leeds Institute of Medical Research, University of Leeds, Leeds, UK; d Department of Medical Physics, Leeds Teaching Hospitals NHS Trust, Leeds, UK; e Division of Cancer Sciences, University of Manchester, Manchester, UK; The Christie NHS Foundation Trust, Manchester, UK; f Laboratory of Radiation Physics, Department of Oncology, Odense University Hospital, Odense, Denmark; g Department of Oncology, Aalborg University Hospital, Denmark; h Department of Oncology, Herlev and Gentofte Hospital, University of Copenhagen, Herlev, Denmark; i Department of Oncology, Aarhus University Hospital, Denmark; j Department of Clinical Medicine, Faculty of Health Sciences, Aarhus University, Denmark; k Department of Oncology, Vejle Hospital, University Hospital of Southern Denmark, Vejle, Denmark; l Danish Centre for Particle Therapy, Aarhus University Hospital, Department of Clinical Medicine, Aarhus University, Denmark

**Keywords:** Reirradiation, workflow, radiotherapy, national guidelines

## Abstract

**Background and purpose:**

Reirradiation is becoming more frequent in clinical practice. However, workflows and practices vary widely between clinics, as general guidelines are scarce or lacking in practical detail. This paper presents comprehensive national Danish consensus recommendations covering all steps of the reirradiation workflow. The aim is to standardise and improve reirradiation treatment quality and provide guidance for much-needed large-scale clinical trials.

**Methods:**

An expert panel was formed comprising physicians, clinical physicists, and clinical researchers from all Danish radiotherapy centres. An in-person 2-day workshop was followed by multiple online meetings. Recommendations were based on expert consensus, supported by review of existing literature, and were reviewed by all Danish Multidisciplinary Cancer Groups before publication.

**Results:**

Reirradiation cases should be designated clearly as such at each workflow step. Review of patient cases at multidisciplinary reirradiation conferences is encouraged. Immobilisation, positioning, and motion management should resemble that of previous treatment(s) as closely as possible. Information on previous dose should be used in planning and evaluation. The degree of complexity (e.g. summation of dose maxima, rigid/deformable image registration, 3D dose accumulation) should reflect the clinical situation as well as the extent/quality of available information. Dose should always be converted to an equieffective dose before summation. Daily image-guidance and regular evaluation of delivered dose are recommended. We provide guidance on quality assurance of dose mapping and guidelines for clinical reirradiation trials.

**Interpretation:**

We present national consensus guidelines for site-independent reirradiation treatment workflows. The guidelines have been approved by the site-specific Danish Multidisciplinary Cancer Groups.

## Introduction

Irradiation of new or recurrent cancers in previously treated areas has been used only sporadically, primarily due to the risk of severe side effects, but has gained traction with the advent of highly targeted and conformal radiotherapy techniques. With a growing number of patients benefiting from improved cancer care, there is an increase in patients experiencing locoregional recurrence or developing a new cancer [1–3]. This highlights the need for effective local treatment options, with reirradiation presenting a promising approach [4, 5].

Reirradiation treatment practices are highly heterogenous, with varying recommendations for patient selection criteria [6–9]. However, as reirradiation has been linked to high rates of severe and even lethal toxicity in previous reports [5, 10–12], the risk of severe toxicity should be considered as part of the patient selection [6, 13–18]. Additionally, the reirradiation volume should be limited, and irradiation of elective volumes may not be feasible [18–20]. Individual patient evaluation and management are essential to minimise the risk of potentially debilitating side effects. This includes accounting for previously delivered doses when evaluating treatment options [6, 21, 22]. However, individual patient management is complex and involves sourcing information on previous treatment, communication between staff, and cumulative dose assessment to estimate toxicity risk. These factors have been reviewed previously [2, 23–26], but detailed national or international guidelines are scarce.

The European Society for Radiotherapy and Oncology (ESTRO) and European Organisation for Research and Treatment of Cancer (EORTC) provided a set of recommendations for patient selection and management [6], but the practical aspects of radiotherapy workflow were covered only in broad strokes. The UK Royal College of Radiologists has published brief principles for reirradiation treatment, but these are aimed primarily at radiation oncologists [27]. Similarly, a framework for dose evaluation in reirradiation aimed specifically at medical physicists was published by the University of Michigan and forms the basis for reirradiation treatment in other US clinics [2, 25, 28]. Detailed guidance on practical management of the entire reirradiation workflow, placing the medical physics aspects within a multidisciplinary framework, has not been developed in a national setting.

Constraints on cumulative doses are based on heterogeneous data from case reports, small retrospective series, and personal experience [5, 6]. To establish meaningful constraints for cumulative doses there is an urgent need for large clinical trials and prospective studies with high-quality clinical and dosimetric data, especially on cumulative doses to organs at risk (OAR) [5, 6]. However, conducting reirradiation trials is highly resource-demanding and requires comprehensive data on previous treatment(s), reliable dose accumulation, and follow-up [12, 14, 17, 29]. Standardising day-to-day clinical management of reirradiation patients could help facilitate trials and allow systematic collection of routine data for future large-scale multicentre data analyses.

The Radiotherapy group of the Danish Comprehensive Cancer Centre (DCCC-RT) has launched an official multidisciplinary Work Package to focus on reirradiation. Here, we aim to provide guidelines for clinical workflow, dose evaluation, quality assurance (QA), and reporting for reirradiation. These guidelines are independent of treatment site. Additionally, based on these recommendations, we propose guidelines for setting up clinical reirradiation trials.

## Methods and materials

In May 2023, physicians, physicists, and researchers held a 2-day reirradiation workshop to achieve national consensus on practical aspects of the clinical workflow, evaluation of reirradiation treatment plans, use of rigid or deformable dose mapping for dose accumulation, and requirements for setting up clinical reirradiation trials.

Sub-groups were formed to address specific challenges: (1) minimum requirements for safely performing and managing reirradiation treatments, (2) QA of image registration and dose accumulation, and (3) design of clinical reirradiation trials. Starting from these broad problem statements, the sub-groups identified points in the reirradiation workflow that were not sufficiently addressed by *de novo* radiotherapy guidelines and where variations in implementation were seen nationally. These points were then discussed in-depth, first at the workshop and in the following months at further online meetings. Consensus statements for recommendations concerning all steps of the radiotherapy workflow were formulated based on these discussions and literature review. These were discussed with, and endorsed by, the disease-specific Danish Multidisciplinary Cancer Groups (DMCGs) and subsequently distributed to all Danish radiotherapy centres.

In the following text, unless explicitly specified, we use the phrasings ‘should’ and ‘may’ to denote stronger versus moderate recommendations.

### Definition of reirradiation

The ESTRO-EORTC recommendations for patient selection and management defined reirradiation as either type 1, where a geometrical overlap exists between the previous and the current irradiated volumes or type 2, where concerns of toxicity from the cumulative doses are present but there is no overlap with the irradiated volume of previous courses [6]. Importantly, this definition encompasses situations where cumulative volume effects are the main concern; these patients may not historically have been considered ‘reirradiation’. We support this definition.

In this report, all treatments ahead of the re-treatment in consideration are denoted as previous treatments and the treatment in consideration is denoted as the current or reirradiation treatment [30].

## Results

### Clinical workflow

**Figure 1:**
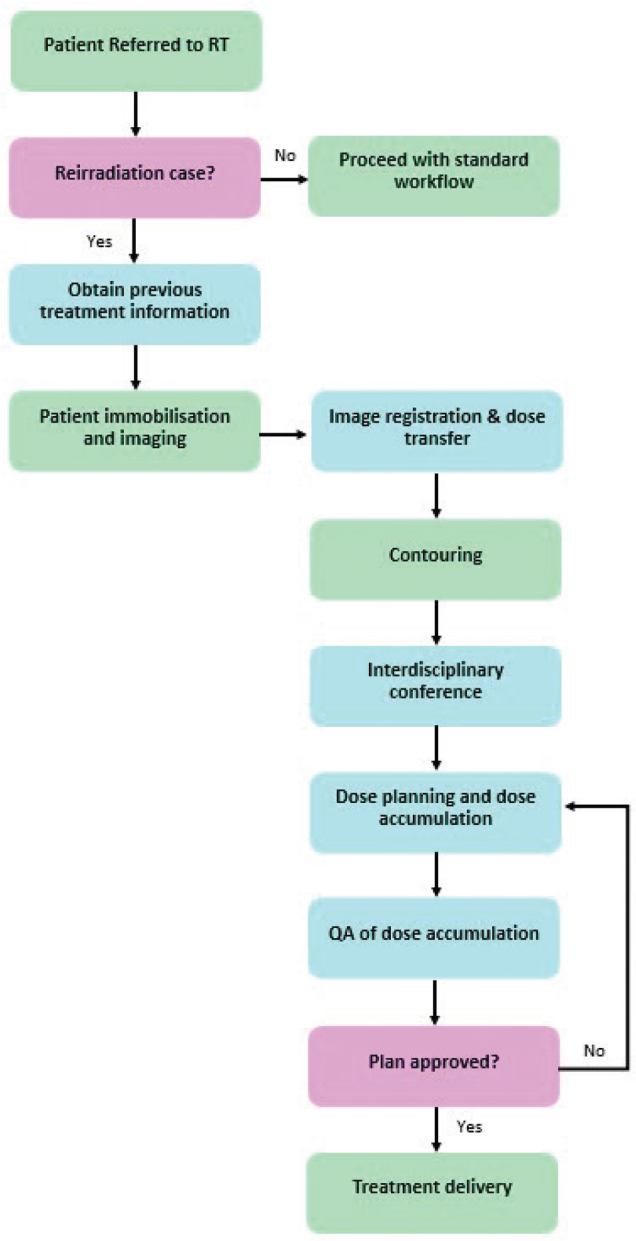
Clinical workflow for patients planned to receive reirradiation. The boxes are colour coded to indicate either a standard part of a patient’s workflow (green), additional steps for reirradiation patients (blue), or important clinical questions (pink).

The following sections contain recommendations concerning all steps of the treatment planning and delivery chain, inspired by the workflow proposed by Paradis et al. [2], see Figure 1.

A key part of a safe reirradiation workflow is documentation and information hand-over. Available information, key decisions, and requests for other staff groups should be documented in a structured and standardised way. This involves identifying patients receiving reirradiation, patient-specific requests such as image registration requirements, requests for OAR prioritisation for treatment delivery, and treatment planning challenges and decisions.

### Patient selection

The decision to re-irradiate depends on several clinical factors such as previously delivered dose, time since previous treatment, other previous (non-RT) treatments, other treatment options (surgery, systemic treatment), side effects from previous treatment(s), comorbidity, adequate organ function, performance status, and preferably a shared decision on risk versus benefit. This very important topic is discussed in detail elsewhere [6, 8, 13–18, 27]. Possible benefits of alternative modalities (e.g. MR linac, brachytherapy, protons) should be considered, as should inclusion in clinical trials.

### Patient referral

Referral for reirradiation should specify the type of reirradiation according to the ESTRO-EORTC guidelines [6] and initiate a dedicated reirradiation workflow. Preferably, this workflow should visually indicate the reirradiation to all staff groups in all succeeding treatment steps.

### Information on previous irradiation

Most patients have received their previous treatment in Denmark, so dosimetric data in digital formats can be transferred using the national infrastructure DCMCollab [31]. Older treatment data may require sending analogue data (e.g. films) via mail or e-mail. Dose may be recalculated with newer and more accurate calculation algorithms if deemed necessary and possible. Likewise, full three-dimensional dose matrices may be reconstructed from analogue data if required. Data from treatments outside Denmark may be impossible to obtain. Treatment planning staff may thus use their best estimate for previously delivered doses based on patients’ reports. All imaging data available for the patient should be requested and taken into account during the reirradiation planning process.

### Patient immobilisation and imaging

Ahead of Computed Tomography (CT) or Magnetic Resonance Imaging (MR), physicians and radiographers should check the patient chart and imaging from all previous treatments. To obtain an optimal image registration for dose accumulation, it is recommended to attempt similar positioning, organ filling, respiratory management technique, and scanning technique if applicable, while also considering patient comfort and the best techniques to minimise toxicity for the current treatment. To this end, immobilisation, positioning, etc. may be discussed with treatment planning staff prior to imaging.

### Image registration and dose mapping

Two types of image registration may be used: either rigid image registration (RIR) or deformable image registration (DIR) [32–36]. RIR applies a uniform transformation of voxels between images, using only translations and rotations. DIR estimates the vector field required to deform the voxels in one image to those in the other based on for example grey tones, structure outlines, or biomechanical models.

In case of complex anatomical changes between previous and current imaging, DIR may not be reliable, adding large uncertainties to the registration and subsequent dose transfer [35, 36]. For these cases, multiple RIRs or DIRs may be performed, positioning the origin and volume of interest of each registration at selected OARs with risk of high cumulative doses. When the registration is not reliable, such as in cases with extreme deformations (e.g. due to surgery or massive atelectasis), voxel-wise dose mapping is not recommended.

As discussed later (‘Dose Evaluation’ section), the detail in which previous dose is considered in reirradiation planning depends both on data availability and clinical need. To optimise workflows, it is important to identify cases in which full 3D dose mapping may not be necessary.

### Quality assurance of dose mapping

The image registration quality should be assessed visually in the region of interest. An in-depth evaluation may be performed as specified in Table 1 [32–38]. Special attention should be given to areas where the dose gradient in the previous dose distribution was steep, as small registration uncertainties can lead to large deviations in dose mapping [36, 39]. Table 1 details methods to assess the impact of DIR uncertainties on the mapped dose.

**Table 1:** Checklist of quality assurance tasks for the deformable dose mapping process, according to national consensus.

Step in dose mapping flow	QA	Priority
**Before DIR**	Is there a large amount of appearing/disappearing tissue in the ROI?	Must
	Is there anything else that makes DIR unreliable in the ROI? (e.g. surgery causing large anatomical changes)	Must
**Previous dose**	Is the previous dose information of sufficient detail/quality?	Must
**DIR assessment**	Use the underlying RIR to identify regions where the DIR should align corresponding anatomical regions.	Must
	If available, use quantitative metrics (e.g. Jacobian determinant) to identify unreliable deformation behaviour.	Should
	Blend images to compare the deformed (moving) image to the current (fixed) image. For example split-window or colormap blending as recommended in [32].	Must
	Visualise the DVF and assess whether it is doing what is expected (no swirls, artificial folds, etc.).	Must
	Assess the deformation of a basic structure (e.g. add a sphere in the region of interest and map it using the registration to assess changes in the space).	Must
	Compare deformed structures to structures contoured on the current image using quantitative metrics such as the 95% Hausdorff distance or mean distance to agreement. If available, structures re-contoured on both images using for example an atlas or neural network may be used. AAPM TG-132 provides suggestions for acceptable deviations from perfect agreement, keeping in mind these are not sufficient in isolation. Notice: All these measures will include delineation uncertainties in the evaluation of the registration, even contours generated by automated solutions.	Must
	Perform several independent DIRs with varying starting point (i.e. a new, independent, RIR for each) or optimisation parameters. Small differences between RIRs and DIRs give an indication of registration stability/consistency. This process can be automated in several commercial software packages.	Should
	Compare anatomical landmarks on the non-deformed and deformed images to assess registration consistency [37]. Depending on the number of points defined this can, however, be quite labour intensive. Further, a certain level of subjective bias is introduced by manually delineating the landmarks.	May
**Assess impact of DIR uncertainties on dose mapping**	Compare deformed dose to rigidly transferred dose and assess if deformation is as expected based on anatomical changes.	Must
	Assess behaviour of DVF in high dose gradient regions.	Should
	Perform at least three independent dose mappings and compare results to ascertain that the dose mapping is consistent. Evaluate visually and assess spread of relevant dose metrics using for example DVH uncertainty bands, standard deviation or inter-quartile range of DVH metrics, or voxel-wise minimum/maximum dose matrices [38].	May
	To assess the deformed dose’s robustness towards uncertainties in rigid registration or inter-fraction shifts in the current treatment, construct the same dose metrics using uncertainty scenarios in which the dose matrix has been shifted rigidly with respect to the current isocentre.	May
**EQD2/BED**	Biological dose correction should always be used. Verify that parameters used are supported by relevant literature.	Must

QA: quality assurance; DIR: deformable image registration, DVF: deformation vector field, RIR: rigid image registration, ROI: region of interest. Recommendation strength is indicated in the right-most column using the same wording as defined in the main text – with one additional level of strength, ‘must’ to indicate steps that are considered absolute.

If full 3D dose evaluation is needed but image registration and dose mapping cannot be performed with acceptable certainty, consider calculating equieffective doses (e.g. EQD2/BED) on the previous scan for the previous irradiation and evaluating relevant dose metrics for each OAR to estimate safe reirradiation dose limits.

### Contouring

The previous treatment(s) should be investigated ahead of target delineation and radiation dose prescription to aid the decision of treatment intent, prescribed dose, and target extent. The latter may differ from de novo guidelines, especially concerning delineation of elective targets due to a different risk–benefit balance in previously irradiated regions. Delineation of additional OARs compared to standard treatments is often required in the high-dose overlap zone.

The quality and validity of image registration may be visualised by comparing OARs delineated on both scans and mapped to the current treatment imaging. This requires consistency in outlining between previous and current treatment.

Targets and OARs should preferably be contoured according to national or international reirradiation guidelines [14, 40–43]. As reirradiation cases seldom resemble ‘standard’ patients as referred to in guidelines and atlases, the use of multimodality imaging and rigorous peer-review of delineations should be considered.

### Interdisciplinary review

All patient cases should receive multidisciplinary review, involving all staff groups involved in treatment planning (dosimetrists, physicists, and physicians), and based on structured local guidelines. Patient-specific compromises discussed at the conference should be documented.

### Treatment planning

Previous treatment, and whether the current treatment represents a reirradiation case, should be noted in the radiotherapy planning records. This may include identifiers in plan name or markers on the record & verify/patient information management system. This is to clearly denote that previous dose must be included in plan evaluation and to facilitate later identification of reirradiations for quality control and research.

**Figure 2:**
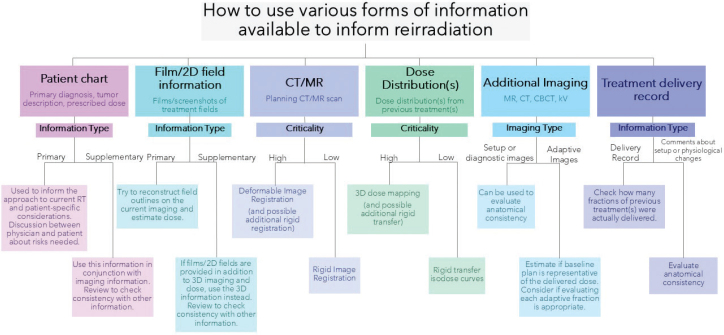
Decision-making flowchart describing how to use information from previous treatment(s). The flowchart is broken down into subsections that describe how to use the given information. A suggested use for the information is provided based on whether it is primary (main/only available information) versus supplementary or high versus low criticality.

**Figure 3:**
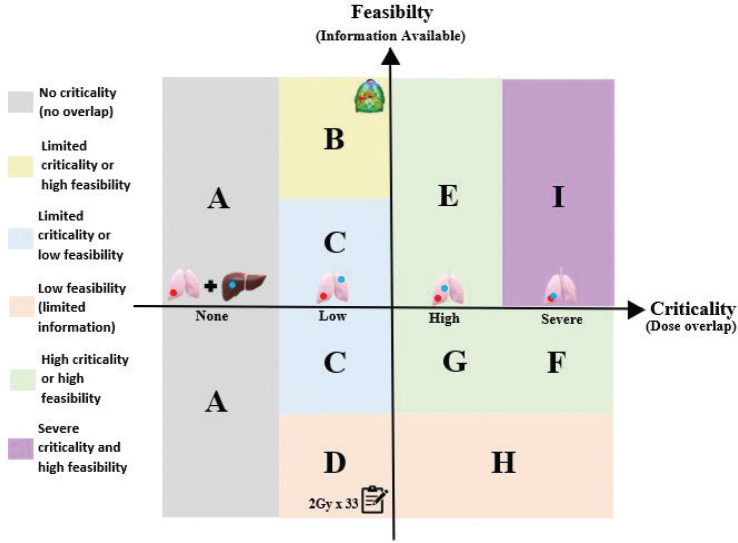
Sectional representation of dose evaluation based on feasibility and criticality. The boundary between scenarios (A and B), (A and C), and (A and D) is the boundary where there is a low dose overlap between the previous treatment and the current treatment volume. The boundary between (B and E), (C and F, C and G), and (D and H) is the transition between low dose overlap between previous and current treatment, to high dose overlap within a critical organs at risk (OAR). The boundary between (F and I) is defined by increasing criticality of the dose overlap. This could be a direct overlap of high doses between the previous and current target, potential for overdosing serial OARs, or an OAR that is highly important for an individual patient.

The dose distribution(s) from previous plan(s) should be considered when creating the dose plan for the current treatment. The level of complexity in this process depends on available information, clinical needs, and treatment planning software (see Figures 2 and 3). Evaluating cumulative doses involves rescaling past and present dose distributions to equieffective doses, a feature currently not supported for optimisation by commercial treatment planning systems [44]. A simple and conservative approach may be to define a custom OAR near-maximum dose constraint (e.g. D_2%_) based on near-maximum dose to the same OAR from previous treatments. This, however, often overestimates cumulative doses [45]. If a 3D dose matrix of the previous treatment(s) and satisfactory RIR/DIR registration(s) are available, the previous dose matrix may be mapped and isodose volumes of interest may be used in optimisation [46].

### Dose evaluation

It is recommended to consider each reirradiation patient on a case-by-case basis. Two primary factors are used to guide the minimum necessary evaluation:

1. Feasibility
  - Time taken to perform the required task versus available time/resources
  - Information available from previous treatment(s) – 3D imaging, treatment plans, 3D doses, films, medical records, prescribed dose, etc.
2. Criticality
  - Overlap with previous target (low or high dose)
  - Previous dose to an OAR
  - Comorbidities, side effects from previous treatment, surgery, systemic treatment, and patient preferences

To aid the decision-making process, a flowchart is presented in Figure 2. This emphasises how available information can guide the reirradiation process and how critical each type of information is in evaluating cumulative doses.

Possible scenarios combining different degrees of feasibility and criticality are shown in Figure 3. The lower left corner represents a case with low feasibility and criticality, for example a case with only dose prescription available and no (or very low) dose overlap. The upper right corner represents a case with high feasibility and criticality, for example a case with 3D dose distributions available and high-dose overlap within critical OAR. Recommendations corresponding to these scenarios including example cases are provided in Table 2.

**Table 2:** Recommendations for dose evaluation in the various scenarios depicted in Figure 3.

Group	Information available	Recommendations
**No overlap with previously irradiated tissue**		
**A**	Any information about prior treatment.	Confirm the previous treatment is not in the vicinity of the new one.
**Low dose overlap between previous and current treatment area**		
**B**	CT-scan + 3D dose distribution	Import the previous plan(s) and transfer isodose curves rigidly.
**C**	Any images 2D or 3D, information about fields	Add a conservative margin around the previously irradiated area and perform some simple hand calculations.
**D**	Patient records: Prescribed dose, treatment area, and treatment technique	As a minimum confirm the previous treatment area and prescription dose.Any other assessment of overlap will depend on the exact information available.
**High dose overlap within a critical OAR**		
**E**	CT-scan + 3D dose distribution	Perform DIR + 3D equieffective dose correction for dose accumulation, provided this is technically feasible.
**F**	Any images 2D or 3D, information about fields	Attempt reconstruction of fields and dose on current imaging. If deemed reliable, perform biological rescaling and 3D dose accumulation.
**G**	Patient records: Prescribed dose, treatment area, and treatment technique	Estimate max point dose with equieffective dose correction (based on prescribed dose).
**H**	Limited information on prior treatment	As essential information is missing, any treatment decisions will require shared decision-making between the physician and the patient about the potential risks of reirradiation.
**Severe risk of fatal/debilitating toxicity**		
**I**	CT-scan + 3D dose distribution	Perform DIR + 3D equieffective dose correction for dose accumulation.

For all recommended actions, the uncertainties inherent in the process and those additionally created by for example large anatomical changes must be taken into consideration.

OAR: organs at risk; DIR: deformable image registration.

### Equieffective dose rescaling

To evaluate cumulative doses across multiple treatment courses irrespective of the fractionation schemes used, doses must be rescaled to equieffective dose (using model parameters supported by relevant literature) before dose accumulation [47]. The α/β values indicating sensitivity to fractionation effects in the linear-quadratic formalism are uncertain for most tissues and differ for early and late adverse effects. For tumours, a range of α/β values, depending on pathology, between 0.4 Gy (liposarcoma) and 16 Gy (oropharynx) has been proposed [48]. For the spinal cord, an α/β value of 2 Gy has been widely used, but values as low as 0.87 Gy have been published [49–51]. A single agreed-upon α/β (early/late) value for each tissue would aid generation of comparable equieffective dose metrics and matrices between centres. As α/β values are likely heterogenous even within a single tissue type, and depend on the endpoint under consideration, different worst-case α/β values may be used for robust cumulative dose evaluation.

Each treatment dose distribution should be individually rescaled to an equieffective dose before dose summation. Even if both the previous and current treatments have prescribed target doses of 2 Gy per fraction, the dose per fraction to OARs differs from 2 Gy.

Very limited data are available on tissue recovery after radiotherapy, with some studies suggesting organ-specific recovery over time [52, 53]. Sparse data from animal models exist for example for brain, spinal cord, bladder, and breast [52, 53]. Robust data on recovery in for example heart, vessels, bronchi, oesophagus, and lungs are lacking [52, 53]. There is no agreement on the tissue recovery factor (TRF) or its time-dependence for each OAR. Some studies suggest at least 6 months between treatments for 25% tissue recovery for the spinal cord [49, 50], while for many OARs no (or only anecdotal) data are available. A consensus on how and when to apply organ-specific TRFs could strengthen the consistency of reirradiation treatments [2].

### Treatment delivery

Daily image guidance with set limits for daily setup uncertainty should be performed. Clear instructions on which structures should be prioritised in the image match, and OARs that may be at risk of over-dosage if uncertainty limits are not respected, should be clearly indicated to treatment staff.

In case of systematic anatomical changes, offline or online adaptation should be performed to secure optimal target coverage and avoid OAR over-dosage [54–57] This is especially important if:

- The image registration used for reirradiation planning is uncertain, for example when large anatomical changes have occurred between previous and current scans
- The transferred dose is uncertain, for example due to steep dose gradients in regions of higher registration uncertainty
- Large anatomical inter-fraction changes are observed based on daily imaging.

Tighter action limits to trigger adaptation compared to de novo treatments will generally be appropriate. Dose evaluation for adaptation may be performed using additional CT/MR scans or may be based on daily image guidance [58–61]. While daily image guidance for setup is strongly suggested, dose evaluations should be performed at least weekly for conventional fractionation and daily for stereotactic treatments. The plan should be adapted in case of over-dosage of critical OARs, or the fraction number may be reduced if replanning is not feasible.

### Reporting

Estimates of cumulative near-maximum equieffective critical OAR doses (e.g. D_2%_) should be reported in patient charts as part of the clinical decision-making and prescription process. Furthermore, any relevant dose-volume histogram (DVH) parameters for OAR should be included if a 3D volumetric dose accumulation has been performed. Information on the image registration and dose accumulation process, such as registration uncertainties, α/β values, and TRFs should be reported as well.

### Clinical reirradiation trials

Clinical trials for reirradiation should include evaluation and recording of toxicity from previous treatment(s) and information on systemic therapy, surgery, and the previous radiation dose delivered. Preferably, trials should use common guidelines for image registration, dose accumulation, QA, and treatment delivery. A comprehensive overview of considerations unique to clinical reirradiation trials is given in the supplementary material. The guideline is aligned with the ESTRO-EORTC consensus statement [6] and serves as an addition to existing guidance for de-novo radiotherapy trials. It should be used in conjunction with guidelines for standard protocol items in clinical trials [62–64].

### National endorsement

These guidelines have been presented to 13 DMCGs (see Supplementary Appendix) covering diagnoses routinely treated with radiotherapy. All relevant DMCGs endorsed the guidelines.

## Discussion

The use of reirradiation is increasing in daily clinical practice [1–3, 6]. However, clinical guidelines concerning treatment workflow, transfer of dose from previous treatment to current, dose accumulation and QA, treatment delivery, and reporting are lacking, possibly impacting treatment decisions. We present a national consensus on reirradiation guidelines for the full planning and delivery process. The guidelines were established based on national workshops and endorsed by the disease-specific DMCGs.

Radiotherapy in Denmark is centralised, procedures are standardised, staff is highly qualified, and the technological resources to deliver state-of-the-art treatments in the reirradiation setting are available [65, 66]. Establishing and distributing these guidelines will aid consistent high-quality treatment across cancer sites and centres nationally and may provide guidance internationally. Standardisation of reirradiation will ensure equity in treatment and benefit all reirradiation patients. Previously published surveys of reirradiation clinical practice have demonstrated huge variation between clinics, for example in collecting previous data, image registration technique, dose accumulation, OAR constraints, and tissue recovery [23, 24, 28].

As outlined by Paradis et al*.* [2], the clinical reirradiation workflow adds several steps to the standard radiotherapy workflow. One of the major elements discussed in Ref. [2] is the collection of previous dosimetric data, a step strongly recommended in the ESTRO-EORTC consensus statement [6]. In Denmark, 3D data is available for the vast majority of patients treated within the last 15 years, that is where treatment was planned based on 3D imaging and with records in modern data formats, and may easily be transferred between clinics using the national dosimetric repository DCMCollab, enabling full 3D dose accumulation [31]. In other countries, data transfer may rely on co-operative agreements between clinics, often complicated by data protection legislation [24]. A second important element is the recommendation for implementation of interdisciplinary reviews facilitating discussions between dosimetrists, physicists, radiation therapists, and physicians [2, 6]. This ensures that all staff groups are kept informed on potential issues for each individual patient referred for reirradiation, thereby securing optimal treatment. A French survey reported that only half of the surveyed clinics had multidisciplinary conferences for reirradiation cases [24]. The presented guidelines emphatically encourage multidisciplinary reirradiation conferences and aim to provide broad guidance for all staff groups involved in the full reirradiation workflow.

In retrospective studies as well as in clinical practice, detailed assessment of cumulative radiation doses from multiple radiotherapy courses is often lacking due to limited access to reliable tools for accurate dose summation [6, 35]. Previous studies have demonstrated significant inter-centre variation in cumulative dose evaluation, improved by consistent use of image registration for 3D dose mapping [67]. Transferring dose from the previous treatment to the imaging used for reirradiation planning can be complicated by anatomical changes between treatments, making QA of registrations crucial [32–37]. In contrast to Paradis et al*.*, these guidelines encourage use of DIR for dose mapping whenever feasible and anatomically reliable. A careful assessment should always be made to ensure that full 3D dose accumulation (whether deformable or rigid) is required and appropriate for each given scenario, and that the inherent uncertainties in the dose mapping process are acceptable [32–37].

Routine use of DIR is dependent on further software advances, including functionality to determine a deformation’s reliability in a quantitative and automated manner [68]. Preferably, uncertainties in the deformation should be provided per voxel and organ. Additionally, DIR algorithms should be able to flag unreliable deformations based on quantitative parameters such as Jacobian determinants or deformation vector field curls to alert users that dose accumulation may not be appropriate. The DIR software should further contain functionality to calculate and visualise statistics on multiple dose-deformation runs (DVH uncertainty bands, voxel-wise min/max doses, inter-quartile range, and similar) to facilitate the assessment of consistency in dose deformation. Comprehensive toolkits for these functionalities are still lacking in commercial software, despite being highly demanded [68].

A unique aspect of reirradiation is the need to involve radiobiological considerations (equieffective dose rescaling or application of tissue recovery) in clinical decision-making [6]. The precise application of radiobiological considerations might considerably impact clinical decisions, maybe even more than image registration and dose mapping. Thus, research in application of radiobiological considerations in a reirradiation setting is highly desirable. Optimisation of the full, biologically corrected, sum of previous and current doses is, as of now, not possible in commercial treatment planning systems, although it is being investigated [69].

Clinical trials on reirradiation are urgently needed to establish safe dose constraints for OARs in the reirradiation setting and guide treatment decisions. Guidelines for de-novo radiotherapy trials have been established and are widely used [62–64]. Reirradiation trials require further considerations: Data collection should include clinical and dosimetric data from previous treatment and the intervening time to current treatment, as well as detailed classification of local recurrence from previous or current cancer. Additionally, the treatment may involve different diagnoses, for example breast cancer as previous and lung cancer as current treatment, which requires different treatment strategies and follow-up. The guidelines presented here may help build a foundation for upcoming reirradiation trials.

In conclusion, guidelines and recommendations for clinical workflow, dose evaluation, QA, and reporting for reirradiation have been established based on a national consensus among Danish oncologists and physicists. The guidelines have been endorsed by the DMCGs. The guidelines are independent of treatment site or primary diagnosis and can be used by all cancer groups. Additionally, guidelines for conducting clinical reirradiation trials have been proposed.

## Supplementary Material



## Data Availability

Not applicable.
