# Supplementary material for: Clinical workflow for reirradiation: national consensus recommendations on imaging, treatment planning, dose accumulation, and treatment delivery
Source: Acta Oncol. 2025 Jul 24;64:43567. doi: 10.2340/1651-226X.2025.43567 (PMC12305692; doi:10.2340/1651-226X.2025.43567)
Supplement: Supplementary file 1 [file AO-64-43567-s1.pdf]

Supplementary material has been published as submitted. It has not been copyedited, or typeset by Acta Oncologica

# Supplementary material

## Appendix

Danish Multidisciplinary Cancer Groups endorsing this guideline:

DACG – Danish Anal Cancer Group

DABLACA – Danish Bladder Cancer Group

DAHANCA – Danish Head And Neck Cancer Group

DaProCa – Danish Prostate Cancer Group

DBCG – Danish Breast Cancer Group

DCCG – Danish Colorectal Cancer Group

DECG – Danish Esophageal Cancer Group

DGCG – Danish Gynecological Cancer Group

DLG – Danish Lymphoma Group

DLGCG – Danish Liver and Bile Duct Cancer Group

DNOG – Danish Neuro-Oncolical Group

DOLG – Danish Oncological Lung Cancer Group

DSG – Danish Sarcoma Group

***Table S1:*** *Summarised overview of the national consensus recommendations for reirradiation workflows.*

### *Guidelines for clinical studies on reirradiation*

This document aims to provide guidelines for setting up clinical trials on reirradiation. The document is based on a national workshop on reirradiation and dose accumulation with the participation of medical doctors, physicists, and researchers from all Danish radiotherapy centres. The intent is to ensure standardised collection and reporting of data. It is meant to complement guidelines on clinical trials for radiotherapy of de novo cancers; thus, standard procedures will not be included in this document. The document includes topics of relevance for reirradiation treatments. For each section, a few recommendations are listed as bullet points. The decisions should be adapted to resources and clinical practice at each centre.

**General considerations:**

In many aspects, the general considerations for a reirradiation trial do not differ from that of any other trial.

It may be valuable to include a screening log. Patients considered for reirradiation are often very heterogeneous even within the same diagnosis, and reasons for not being included in the trial can be more diverse than in the de novo setting. Consider if it is relevant to include these patients not enrolled in a prospective registration protocol instead.

Patient involvement and shared decision-making tools are often relevant for reirradiation, as the chance of benefit versus the risk of side effects are balanced differently than in the de novo setting.

The content and schedule of the trial follow-up should reflect that tumour response and side effects are different than in the de novo setting. Consider if the standard PROs/CTCAEs reflect the side effects for reirradiation or should be extended, and if different scan modalities or different intervals than those used in the de novo setting should be used. Consider including translational studies in the trial.

Reirradiation trials will often be run in a national or international setting. Consider if all centres should provide the reirradiation or if there should be a centralised function within the trial.

- Keep a screening log
- Practice shared decision making
- Content and schedule of follow-up should be reirradiation-specific
- Consider including translational studies
- Consider centralization of reirradiation

**Pre-treatment protocol considerations**

Reirradiation is defined according to the ESTRO-EORTC consensus guidelines on definition, reporting, and clinical decision-making for reirradiation [Andratschke2022] as radiotherapy delivered either to a previously irradiated volume (irrespective of concerns of toxicity) or in which the cumulative dose raises concerns of toxicity. Two different scenarios are possible: reirradiation type 1 is any new course of radiotherapy that has geometrical overlap with the irradiated volume of previous courses, and reirradiation type 2 is a new course with concerns of toxicity from the cumulative dose without overlap of irradiated volumes (Andratschke2022). These definitions should be used in a prospective clinical protocol.

- Use ESTRO-EORTC consensus guidelines for the definition of reirradiation.

**Previous radiotherapy**

Information on the previous radiotherapy course(s) should be collected. This includes dose prescription, fractionation, and the delivered number of fractions. The treatment modality and treatment technique should be reported; preferably including information on image guidance, use of adaptive radiotherapy, inclusion of respiratory information and management (e.g., 4DCT, DIBH), bladder filling protocol, and any other specific techniques. The details on actual treatment delivery should be obtained to aid the uncertainty estimate. Moreover, previous CT images and 3D dose

distribution should be collected if available to facilitate evaluation of cumulative doses and establishment of dose-response relationships.

Based on the patient chart, the response to the previous radiotherapy course should be collected for the tumour. Similarly, for the organs at risk, any persistent or severe toxicity in the anatomical region to be reirradiated should be noted. It should be defined in the protocol which toxicity data to collect, i.e. state relevant organs at risk and toxicity grades. The time elapsed since the previous irradiation(s) should be recorded and the protocol may set a limit for the minimum timespan between irradiations. A pragmatic approach for the minimum timespan is recommended, hereby aiming for patient-individualised management.

- Collect information on immobilization, image guidance, respiratory management, previous 3D dose distribution & imaging (if available).
- Collect information on severe toxicity and tumour response to previous radiotherapy course.
- Time span since previous radiotherapy should be obtained.

**Other previous treatments**

Data on previous treatments that are related to the disease in question or could affect the current reirradiation course should be collected.

Response and side effects to previous antineoplastic systemic therapy could affect reirradiation. This regards both concomitant, neo-adjuvant, and adjuvant systemic therapy.

Previous surgery in the area for reirradiation could affect the co-registration of scans, estimation of cumulative doses, patient positioning possibilities, etc. Also, addition/removal of implants or tissue reconstructions could affect the feasibility of some scanning and radiotherapy techniques/modalities (e.g. MRI or proton therapy in the case of dense metal implants).

- Collect information on previous systemic therapy and timing.
- Collect information on previous surgery that could affect any part of the RT planning and evaluation chain.

# Endpoints

The endpoints specified in the trial should take the complexity of performing a reirradiation trial into account. Patients with different histologies, diagnoses, and life expectancies may be included to facilitate a trial with a sufficient number of patients. As only sparse data exist for reirradiation, the data acquisition may be aligned with other reirradiation trials to pool data after trial closure. The oncological outcome measures should reflect the expected outcome, e.g. tumour control vs. complete response or the rate of severe toxicity.

- Consider aligning data acquisition with other reirradiation trials.

**Trial Quality Assurance**

In a multi-centre trial, pre-trial quality assurance (QA) increases standardisation and adherence to protocol definitions and requirements. Specifically for reirradiation, consensus on target definition, relevant organs at risk, and dose accumulation are important. Consensus-making can be performed by requiring that all participating centres delineate 3-5 selected clinical cases and similarly perform treatment plans for 3-5 pre-delineated cases. All of these should be based on the guidelines in the clinical protocol. The completed cases should be reviewed centrally. In case of centre-wise deviations, feedback should be provided, and corrections should be made. For the dose accumulation, detailed guidelines and requirements should be provided, and dose accumulation should be performed in 3-5 cases representative of the trial population. After approval, each centre should be responsible for internal training of relevant personnel to ensure that all staff follow the guidelines. The trial QA should be adapted to available clinical resources.

After trial initiation, selected cases can be discussed at meetings between participating centres. A central review of selected on-trial cases can be used to secure continued adherence to the protocol.

- For pre-trial QA, consider requiring that all centres delineate 3-5 clinical cases, perform treatment plans for 3-5 cases, and accumulate doses for 3-5 cases.

**Patient and tumour characteristics**

When determining inclusion and exclusion criteria, consider adding pragmatic criteria rather than very specific criteria, e.g. on timespan since previous radiotherapy, treated volumes, and side effects from any previous treatment.

Consider if biopsy verification of recurrence is always needed, as this can be difficult in a previously irradiated or resected area. In some instances, "unequivocal recurrence" on diagnostic imaging may be sufficient.

Specify if the reirradiation trial should include patients being reirradiated for the same diagnosis (including cancer recurrence) or all patients formerly irradiated in the specific anatomical region (e.g., thorax or abdomen) irrespective of specific cancer diagnosis. Likewise, specify whether new primary tumours, metastases, and recurrences may all be included in the same protocol (or not).

Specify the intent of the reirradiation (post-operative, definitive, neo-adjuvant, palliative). Furthermore, define if both reirradiation type 1 and 2 can be included in the trial.

- Consider using/adding pragmatic inclusion/exclusion criteria.
- Define the intent of the reirradiation.

**Imaging**

The minimum requirement for imaging for the reirradiation treatment should be noted. Also consider if alternative imaging techniques and modalities could be beneficial for target definition, e.g. specific MRI sequences, PET-CT scans with contrast delays, etc. During imaging and treatment for reirradiation, patient positioning similar to that of the previous treatment may be preferable to minimise uncertainties in dose accumulation. Similarly for respiratory management, bladder filling protocols, etc. Standard or patient-individual fixation can be used.

- Consider using patient positioning similar to that in the previous treatment course.

- Consider alternative scan modalities.

**Delineation**

Guidelines for delineation should state a minimum list of organs at risk (OAR) to delineate and provide guidance on how the OAR should be delineated, including e.g. craniocaudal extension. An atlas may be of help.

A standard target delineation guideline should be provided including margin for CTVs, creation of iCTVs, etc. When to include or not to include elective volumes should be defined.

If compromises in target extension e.g. for the elective volume are accepted to reduce the irradiated volume, these should be described.

- Include a list of organs at risk to delineate.
- Add guidance for acceptable compromises in target delineation.

**Treatment planning**

3D dose planning should be performed taking the previous dose distribution into account. Guidelines for cumulative equieffective (EQD2 or BED) dose constraints for the OAR should be given, preferably with minimum first-priority constraints being mandatory to fulfil and additional constraints being preferable but optional to fulfil. The constraints may be based on the best available data from literature or pragmatic decisions, allowing the treating physician to make patient-individual decisions. For curatively intended treatments, a specification of the minimum acceptable dose for the GTV, CTV, and PTV should be stated.

- Add equieffective cumulative dose constraints
- Aim for pragmatic dose constraints to be optional for the treating physician to respect.
- Specify the minimum acceptable dose for the GTV, CTV, and PTV.

**Dose accumulation/summation**

In the case of reirradiation type 1 or 2, it is recommended that availability of previous dose distribution(s) is an inclusion criterion. The scans and 3D dose distribution(s) of previous treatment(s) should be acquired, and the dose distributions accumulated. The guidelines for dose accumulation must provide minimum and recommended requirements for image registration, recalculation of the previous plan with an advanced dose calculation algorithm, equieffective dose rescaling and calculation of cumulative dose, and recovery factors. In case of anatomical changes between the previous and current treatment imaging, prioritisation of regions which are required to have the best match should be stated, e.g. for reirradiation near the spinal cord a high precision of the match is typically recommended at the position of the cord. Guidelines on handling and reporting substantial anatomical changes between previous and current imaging, e.g. due to surgery, organ motion, deep inspiration breath hold versus free breathing, or growth in the case of children, should be provided. For the trial, a workflow for quantitative uncertainty analysis may be considered as well as a workflow for calculation of uncertainty scenarios.

- Acquire the treatment plan imaging and 3D dose distributions for the previous treatment(s).

- Describe requirements for image registration, biological recalculation, use of recovery factors, handling of large anatomical changes, and estimation of uncertainty.
- Describe the prioritisation of regions which are required to have the best match on any image registration.

**Treatment delivery**

For treatment delivery, the applicable delivery and image guidance modalities should be stated. The patient should be treated using daily imaging and plan adaptation should be made in case of deviations above pre-set limits. Instructions on tolerances for regions with a risk of overdosage of OAR due to reirradiation should be communicated to the radiation therapists, e.g. as a note or by delineating the OAR accompanied by a pre-defined tolerance. Motion management should follow the choice made for treatment plan imaging.

- Use daily pre-treatment imaging.
- Consider providing tolerances for deviations seen in regions with a risk of over-dosage of OAR.

**Follow-up**

The follow-up may be aligned with other reirradiation trials to pool data after trial closure. Reirradiation may result in excessive side effects and the risk of recurrence in this patient group is increased. Thus, patient-reported outcome measures (PROM), quality of life (QoL), and toxicities specifically related to reirradiation should be scored. Note that these can be different from those of de novo irradiation. Also, consider collecting data on the patient's perspective on received reirradiation in the follow-up (e.g. decision regret measures). For the toxicity evaluation, it should be defined when to stop toxicity registration (e.g. at progression or when initiating other treatments) and which types of toxicity to evaluate and record. Consider recording only grade 3-5 toxicities. A minimum requirement for follow-up imaging should be stated and may be based on the patient group for irradiation.

- Collect PROM, QoL, and toxicity data related to reirradiation.
- Consider aligning data collection with similar reirradiation trials.

**Supportive care**

Consider if supplementary supportive care is needed within the trial setup. This could include geriatric assessment up front, referring to specific units for late effects, support on sexual health or nutrition, or specific rehabilitation.

- Consider if geriatric assessment or other rehabilitation measures may be helpful.

**Data storage and reporting**

Images, delineated structures, information on image registration (e.g. deformation vector field), and dose plans for the previous and current treatment should be uploaded to centralised dose plan databases. Additionally, the mapped 3D dose from previous treatment image to current image and/or the cumulative 3D dose should be collected. However, the values for max/near max/mean/minimum

target and selected OAR doses to report after trial closure may be predefined for extraction. Clinical data should be stored in an electronic case record form database. Information on the previous radiotherapy should be collected. This includes information on treatment outcomes and severe toxicity.

If large anatomical changes are seen between the previous imaging and the imaging used for reirradiation, this should be noted. Furthermore, an estimate of the uncertainty in the dose accumulation may be given. If first-priority constraints in the protocol cannot be respected, the treatment plan may be accepted by the treating oncologist and the reason for accepting over-dosage of OAR or under-dosage of target should be noted. All diagnostic and follow-up images should be saved for the reirradiation treatment. Similarly, compromises in target delineation may be noted.

- Upload all images and dose plans for previous and current treatment plans.
- Store information on treatment outcome and severe toxicity for previous treatment in a centralised database.
- Store information on image registration, dose accumulation and uncertainty, as well as compromises made during the delineation and treatment planning of the reirradiation plan.

Table S1: Overview of the consensus recommendations for a clinical reirradiation workflow.

| **Step in clinical workflow** | **Recommendation** |
|---|---|
| Referral | Clearly denote as reirradiation to all following steps. |
| Immobilization and imaging | Review prior scans and, if feasible, aim at similar immobilization, positioning, motion management, and scan technique. |
| Image registration and dose mapping | Registration and dose mapping technique should reflect feasibility (available time, quality of information, anatomical changes) and clinical need. |
| QA of registration and dose mapping | Verify sufficient quality of prior dose matrix.<br>Registration (especially) DIR may be unreliable if large anatomical changes occur between prio and current scans.<br>Pay special attention to DIR uncertainties in regions with high prior dose gradient.<br>Compare rigidly and deformably transferred doses. |

| | |
|---|---|
| | Visualize deformation vector field.<br>If feasible, perform several independent dose mappings to gauge consistency and extract statistical metrics (e.g. DVH bands or voxel-wise dose metrics).<br>Robustness may be evaluated using rigid shifts of the dose matrix. |
| Contouring | Review prior contours. Additional OAR contours may be necessary. Target contours may differ from de-novo RT setting. Use contouring guidelines, if available and appropriate. |
| Interdisciplinary conference | Interdisciplinary reirradiation conferences are encouraged.<br>Up-to-date information should be available to all relevant staff. |
| Dose planning | Clearly identify plan as reirradiation.<br>Consider prior dose during planning. |
| Dose evaluation | Consider on case-by-case basis. Necessary degree of evaluation complexity depends on feasibility and criticality. |
| Biological correction | Always convert dose to equieffective dose (e.g. EQD2 or BED).<br>Model parameters (e.g. $\alpha/\beta$) should be supported by literature.<br>Use commonly agreed-upon model parameters for inter-centre comparable planning. |
| Treatment delivery | Use daily image guidance with set limits for setup uncertainty.<br>Give clear instructions for structure prioritization to treating staff.<br>Dose evaluation on anatomy of the day is recommended at set intervals. |
| Reporting | Relevant DVH parameters should be reported in patient charts.<br>Information on registration process, biological correction, and uncertainties should be recorded. |
